# Maxillomandibular advancement for obstructive sleep apnea: a contemporary review

**DOI:** 10.3389/fsurg.2026.1714967

**Published:** 2026-08-10

**Authors:** Martin Kauke-Navarro, Derek Steinbacher, Ali-Farid Safi

**Affiliations:** 1 Department of Surgery, Division of Plastic and Reconstructive Surgery, Yale New Haven Hospital, Yale School of Medicine, New Haven, CT, United States; 2 West River Surgery Center, Guilford, CT, United States; 3 Craniologicum, Center for Craniomaxillofacial Surgery, Bern, Switzerland

**Keywords:** apnea–hypopnea index, cardiometabolic health, longevity, maxillomandibular advancement, obstructive sleep apnea, orthognathic surgery, sleep surgery

## Abstract

Obstructive sleep apnea (OSA) affects up to one billion adults worldwide and is associated with hypertension, cardiometabolic disease, neurocognitive decline, and increased all-cause mortality. While continuous positive airway pressure (CPAP) remains first-line therapy, more than half of patients are intolerant or non-adherent, leaving a substantial population at sustained risk. Maxillomandibular advancement (MMA) is one of the most effective surgical treatments for anatomically driven OSA to reconstruct a functional and patent airway, achieving approximately 80% reduction in the apnea–hypopnea index, durable improvements in nocturnal oxygenation, daytime function, and quality of life. Beyond standard apnea metrics, evidence links MMA-mediated correction of intermittent hypoxia to attenuation of oxidative stress, sympathetic hyperactivation, and cellular senescence pathways implicated in accelerated aging which positions MMA as a potentially disease-modifying intervention with implications for longevity. In this review, we synthesize the contemporary evidence on patient selection, surgical planning, magnitude of advancement, complications, and comparison with alternative therapies, and we identify key research gaps including the absence of long-term cardiovascular endpoints, head-to-head comparisons with hypoglossal nerve stimulation, and MMA-specific cost-effectiveness data. MMA should be considered not merely as craniofacial corrective surgery but as a definitive, healthspan-relevant treatment for selected patients with OSA.

## Introduction

Obstructive sleep apnea (OSA) affects 9%–38% of adults worldwide, representing nearly one billion individuals, and has profound consequences on quality of life and overall health (1, 2). OSA is a multifactorial disorder arising from a combination of anatomical and physiological factors that cause recurrent upper airway (pharyngeal) collapse during sleep, resulting in transient, intermittent, hypoxemia and fragmented sleep (3). Risk factors include age, body mass index (BMI) and too narrow upper conductive airways (4, 5).

Intermittent airway obstruction produces the classic symptoms of snoring, nocturnal awakenings, unrefreshing sleep, and excessive daytime sleepiness, which are linked to impaired quality of life, adverse health outcomes and even increased risk of traffic accidents (6, 7). Chronic untreated OSA promotes sympathetic hyperactivation, contributing to hypertension, insulin resistance, cardiovascular mortality, and neurocognitive decline (1, 4, 6, 8, 9). In fact, long-term data demonstrate that sleep-disordered breathing (SDB) significantly increases all-cause mortality (5, 10). For example, in the Wisconsin Sleep Cohort, the adjusted hazard ratio for all-cause mortality in patients with severe SDB (AHI ≥30) compared to no SDB was 3.0 (95% CI, 1.4–6.3) (10).

In plastic surgery, few procedures unite aesthetic optimization with durable functional gains and potential impact on general health. This brief review evaluates maxillomandibular advancement (MMA) for anatomically driven OSA in selected patients and synthesizing evidence that airway expansion improves nocturnal oxygenation, reduces hypoxic burden, and yields sustained benefits in blood pressure, cardiometabolic risk, daytime function, and quality of life.

### Management of OSA

Conservative management typically involves weight loss and positive airway pressure therapies such as CPAP (8). Although many OSA endotypes exist, some patients are primarily affected by anatomical factors such as obesity, craniofacial anomalies with narrowing of the airway space. Others exhibit non-anatomical mechanisms including impaired ventilatory control or reduced responsiveness of upper airway musculature (4, 11).

Aside from more general optimizing interventions (e.g., lifestyle modification, bariatric surgery), conservative management mainly consists of positive airway pressure (PAP) therapy including CPAP therapy as well as the use of mandibular advancement devices (MAD). Surgically, a wide range of surgical procedures are available, designed to improve airway patency (2, 12). These include nasal surgery (turbinectomy, septoplasty), tongue base procedures (resection, genioglossus advancement), palatal procedures (tonsillectomy, palatopharyngoplasty), and in some patients with complete lateral pharyngeal wall collapse on drug-induced sleep endoscopy (DISE) or with clear dentofacial anomalies and intolerance to CPAP or other conservative measures, maxillomandibular advancement (MMA) (12). Historically, MMA was offered only after less invasive options had been tried (as a phase II procedure following phase I such as soft tissue reduction procedures), however given the excellent outcomes, MMA is considered first line surgical options for selected patients in which the skeletal framework is the presumed primary driver of OSA (see below) (11, 13, 14)

**Figure 1:**
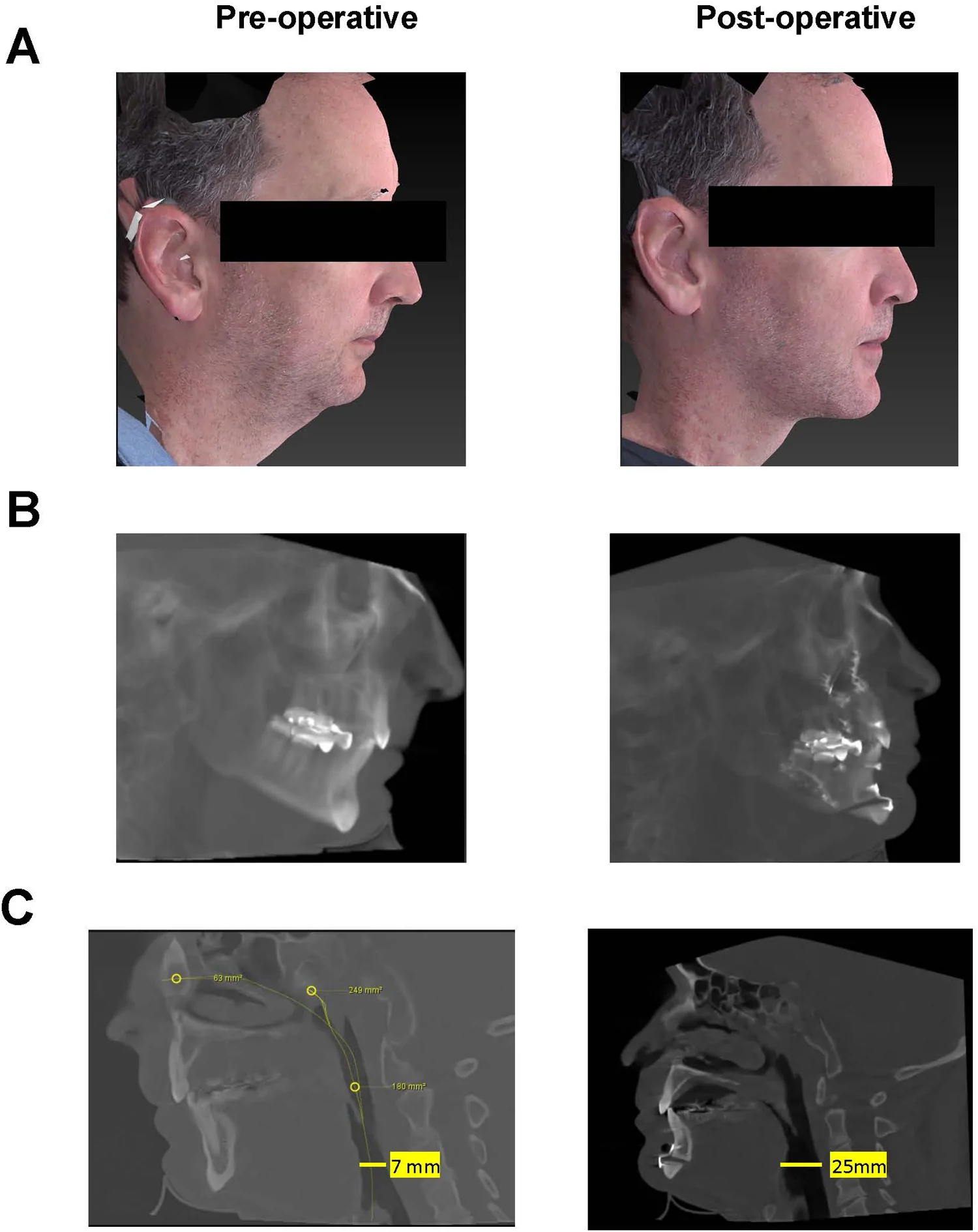
Panel **A** shows side profile photographs of the same individual before and after MMA, with eyes blacked out for privacy. Panel **B** contains corresponding pre-operative and post-operative lateral radiographic scans, highlighting skeletal changes. Panel **C** displays sagittal CT images, with measurements indicating linear airway space increasing from 7 millimeters pre-operatively to 25 millimeters post-operatively.

For patients with craniofacial anomalies and moderate to severe OSA, MMA enlarges the pharyngeal airway through advancement of the maxilla and mandible (typically with counterclockwise rotation of the maxilla), thereby reducing airway collapsibility and correcting anatomical contributors to obstruction (See Figure 1) (1). Systematic reviews confirm that MMA significantly improves OSA outcome metrics, including the apnea-hypopnea index (AHI) and oxygen saturation nadir (14, 15). For detailed review of common indications and considerations see Table 1.

**Table 1:** Patient selection criteria.

Category	Criteria (published in the literature)
Indications to support MMA for OSA treatment	Moderate-severe OSA (AHI ≥15 events/h) in skeletally mature patients with appropriate phenotyping (physical exam + PSG) (47). PAP failure (intolerant, non-adherence, or inadequate adherence) and/or MAD failure after adequate trial (12, 48) Craniofacial skeletal deformity (Class II/III, retrognathia, maxillomandibular hypoplasia) – MMA may be offered as primary surgical therapy (without phase-1) DISE findings unfavorable for soft-tissue or neurostimulation approaches: complete lateral pharyngeal wall collapse or complete concentric palatal collapse (12, 47) Inadequate response to phase-1 (intrapharyngeal) surgery if not a candidate for MMA first approach (12)
Relative indications	Mild OSA (AHI 5–14.9 events/h) with refractory symptoms and concurrent dentofacial deformity warranting orthognathic correction (24, 47, 49) High predicted CPAP non-adherence or patient preference for definitive single-stage surgical treatment after informed discussion of alternatives (including hypoglossal nerve stimulation in eligible candidates) — AASM conditionally supports referral for BMI <40 kg/m² with persistent inadequate PAP adherence (48) Residual OSA following prior upper-airway surgery (e.g., UPPP, genioglossus advancement) per Stanford Protocol (12)
Contraindications	Incomplete skeletal maturation Non-anatomic OSA endotype (predominant central sleep apnea without upper-airway collapse) (12, 47, 49) Uncontrolled psychiatric comorbidity, active substance-use disorder, uncontrolled systemic illness precluding safe general anesthesia, or inability to comply with perioperative protocol Morbid obesity (BMI >40 kg/m²) — relative contraindication given reduced success rate (see below), increased perioperative risk, and higher likelihood of residual OSA; pre-operative weight reduction or bariatric surgery can be recommended first where feasible (12)
Favorable anatomic and outcome predictors	Younger age (<45 years) — Zaghi meta-analysis (*n*=518): younger age was an independent multivariate predictor of greater % AHI/RDI reduction; Holty & Guilleminault meta-analysis (*n*=627) confirmed younger age as predictor of success (47) Lower preoperative BMI (<25 kg/m²)9, younger age (<45), preoperative AHI <44.5 events/h, Preoperative SNB <75°, Maxillary advancement <11 mm (19, 50) Lower preoperative AHI (<45 events/h) — lower baseline AHI associated with higher cure rate, though patients with severe OSA also show the largest absolute AHI reduction (19, 50) Retrognathia with pre-operative SNB <75° on lateral cephalometry — mandibular retrusion predicts favorable response (no difference when looking at SNA angle) (50) Narrow posterior airway space (PAS <8 mm)/retrobasilingual space and superior posterior airway space (SPAS) on cephalometry (non-responders had larger airway segments as measured on lateral cephalogram preoperatively) (19, 47, 50) Greater maxillary advancement (≥10 mm) — degree of maxillary advancement is an independent predictor of surgical success across meta-analyses (19) Counterclockwise rotation of the maxillomandibular complex — maximizes airway expansion while preserving aesthetics (51) Absence of cardiovascular disease and low central apnea index — cardiovascular comorbidity and higher CAI independently reduce odds of favorable response (12)

AHI, apnea–hypopnea index; BMI, body mass index; CAI, central apnea index; DISE, drug-induced sleep endoscopy; MAD, mandibular advancement device; MMA, maxillomandibular advancement; OSA, obstructive sleep apnea; PAP, positive airway pressure; PAS, posterior airway space; SNB, sella-nasion-B point angle; UPPP, uvulopalatopharyngoplasty.

### Diagnostic testing and definitions

Obstructive sleep apnea is diagnosed with polygraphy (PG, home based test) or polysomnography (PSG, in-laboratory testing) which yields the apnea–hypopnea index (AHI). The AHI reflects the number of apnea (complete cessation of airflow ≥10 s) and hypopnea (relative reduction in airflow) events per hour of sleep (2). Patients with more than 15 events per hour—or at least 5 events per hour when accompanied by classic symptoms such as daytime sleepiness, fatigue, choking/gasping, or loud snoring—meet diagnostic criteria for OSA (6, 14, 15).

According to current guidelines, OSA severity is classified as follows (15, 16): Normal: AHI <5 events/h, mild: AHI 5–14.9 events/h, moderate: AHI 15–29.9 events/h, severe: AHI ≥30 events/h. Surgical success is generally defined as a ≥ 50% reduction in AHI or an AHI <20 events/h (15, 17). Polysomnography can also differentiate central from obstructive events: in central sleep apnea, airflow ceases for ≥10 s without respiratory effort, whereas in OSA respiratory effort persists against an obstructed airway (3).

### Beyond AHI: additional metrics

While AHI remains the standard severity marker, additional PSG-derived parameters provide complementary insights (16). The oxygen desaturation index (ODI) quantifies the number of ≥3%–4% desaturation events per hour, while nadir SpO₂ and percentage of time spent with oxygen saturation <90% also capture hypoxic burden (18). Higher ODI and lower nadir SpO₂ are strongly associated with worse disease severity, and some studies suggest desaturation-based indices may better reflect OSA burden than AHI alone (18).

### Anatomical considerations

Airway anatomy alone does not fully predict OSA severity, but certain structural features increase risk (2). These include dysgnathia (Class II or III skeletal relationships or maxillomandibular hypoplasia), increased soft tissue volume of the velum and pharyngeal walls, and heightened airway collapsibility due to reduced muscle tone (2, 11). These factors are potentially modifiable with surgical intervention.

### Rationale and mechanisms of MMA surgery

Maxillomandibular advancement (MMA) can enlarge the posterior pharyngeal airway by moving the mandible and maxilla. MMA has been shown to be effective for both Class II and Class III skeletal anomalies, although patients with Class II anatomy (maxillary retrognathia) often present with more severe baseline AHI and ODI scores (2, 19).

Historically, under the Stanford protocol, MMA was considered a phase II procedure, reserved for patients with persistent disease following phase I surgeries (e.g., nasal, soft palate, or tongue base interventions) (12). However, subsequent studies demonstrate that some patients—particularly those with dentofacial anomalies or complete lateral pharyngeal wall collapse identified on DISE—benefit from proceeding directly to MMA (12).

MMA is now an established option for patients with OSA who fail to improve adequately with other interventions (2, 14). Volumetric imaging consistently shows increased pharyngeal airway space following MMA (11). Technically, the procedure combines a Le Fort I osteotomy with bilateral sagittal split osteotomy (BSSO), repositioning the jaws anteriorly to expand the airway in a single stage. Although less invasive procedures such as isolated genioplasty can modestly improve posterior pharyngeal airway dimensions, their effectiveness in alleviating OSA symptoms remains less certain and continues to be investigated (20).

Large meta-analyses affirm the efficacy of MMA: when success is defined by AHI or respiratory disturbance index (RDI) improvements, MMA consistently achieves high rates of surgical success and is considered a definitive treatment for appropriately selected patients (15).

### Surgical planning and magnitude of advancement

Contemporary MMA is planned by using three-dimensional virtual surgical planning (VSP) based on preoperatively acquired cone-beam CT (CBCT) or multidetector thin slice CT which allows the surgeon to both simulate skeletal movements, predict changes of the airway space and visualize aesthetic changes of the soft tissues. This process was shown to improve outcomes in this population with greater predictability of airway gains and symptom improvement (21). Furthermore, this process allows creation of patient-specific cutting guides and osteosynthesis plates.

The workflow will include planning of a LeFort 1 osteotomy (single or multi-piece) which is combined with bilateral sagittal split osteotomy (BSSO) and counterclockwise rotation of the maxillomandibular complex to maximize both anterior movement of the mandible and optimize pharyngeal airway soft tissue tensioning while limiting negative aesthetic impact of large maxillary advancements (12). Although frequently reported to be at 10 mm advancement, the optimal magnitude of advancement to achieve greatest airway gain, maintain aesthetic relationships and achieve symptom/AHI control has not been precisely defined. One of the issues is where the advancement was measured from – traditionally advancement was measured on 2D lateral cephalograms (measured from incisor or A-point or B-points only). This way of measurement may miss some of the 3D changes of the upper (e.g., CCW) and lower law (e.g., occlusal/mandibular plane angle changes).

However, Holty et al. found that greater degree of maxillary advancement was one of the greatest predictors of surgical success (OR of success 1.97 per 1 mm of maxillary advancement) (19). The patient cohort with surgical cure (AHI <5) had mean maxillary advancements of 9.5 and mandibular advancement of 10.8 mm (19). An early but influential series by Riley and colleagues was among the first to suggest a dose–response relationship between the magnitude of skeletal advancement and surgical success (11). In their cohort of 40 patients, the successful group (*n* = 36) had greater mean maxillary, mandibular, and genioglossus advancements than the unsuccessful group (7.25 ± 1.2 vs. 5.8 ± 1.5 mm; 10.9 ± 2.5 vs. 9.8 ± 2.6 mm; and 13.3 ± 1.8 vs. 12.5 ± 1.9 mm, respectively), with no surgical failures observed in patients receiving larger advancements. Although these between-group differences did not reach statistical significance, the consistent directionality across all three skeletal segments helped establish the principle that adequate magnitude of advancement is a key determinant of MMA outcome. Lastly, the counter clock wise rotation combined with maxillary advancement has also been shown to be significantly associated with airway gains and AHI reduction (13).

### Efficacy of MMA: sleep and physiologic outcomes

Numerous cohort studies and meta-analyses have quantified the impact of MMA on OSA severity (see Table 2).

**Table 2:** Summary of selected articles reporting on the success of MMA for OSA management.

Study (Year)	N, BMI (kg/m2), age (years)	Preop AHI (events/h)	Postop AHI (events/h)	AHI Reduction	Key Findings/ Secondary Outcomes
Systematic Reviews/Meta Analysis					
Zaghi et al. (2016) (14)	518 (mean BMI 33.8, mean age 45.3)	57.2	9.5	∼80%	Most-cited meta-analysis SpO₂ nadir: 70→87%; ESS: 13.5→3.2 Predictors of treatment success: Linear correlation between preoperative AHI values and effective reduction in AHI postoperatively Preoperative AHI 30–60/events/h strongest predictor of surgical cure (lower cure rates if >60 preoperatively)
Camacho et al. (2019) (41)	120 (6 patient populations - mean BMI 28–31.4, mean age 40.7 to 53.8)	Long term 4–8 years (65.8) Very long term >8 years (53.2)	Long term 4–8 years (reduction to 9 in the short term and stable at 7.7 in the long term) Very long term >8 years (8.7 immediately after surgery and increase to 23.1 very long term)	>85% (long-term); ∼57% (very long-term)	Leading Systematic review on long term outcomes (studies with at least 1 year follow up) Increase back to moderate OSA in the very long term noted in this patient cohort
Holty et al. (2010) (19)	627 (22 patient populations – mean BMI 26.9 – 35.3, mean age 36.5–50.3)	63.9	9.5	86%	One of the foundational systematic reviews/meta-analyses to determine predictors of surgical success Predictors of success: younger age, lower preoperative weight and AHI, greater degree of maxillary advancement
Cohort Studies					
Riley et al. (2000) (11)	40 (mean BMI 31.4, mean age 45.6)	71.2 (RDI)	7.6 (RDI)	∼89%	Landmark long-term Stanford cohort; approx. 90% clinical success with MMA “Long-term” follow up (mean: 51 months). Positive correlation between degree of advancement and clinical outcome (greatest RDI reduction with mandibular advancement >12 mm) Demonstrated durability of skeletal airway surgery; major weight gain was only factor compromising long-term outcomes.
Boyd et al. (2015) (28)	30 (mean age 50.5, mean BMI 29.1)	49	10.9	∼80%	Leading long-term prospective two-center cohort study to assess stability of changes >2 years after MMA (mean 6.6 years after MMA) 83% of patients with AHI < 15 events/h Diastolic BP decrease noted (83.7 -> 79) Authors demonstrate that AHI reduction is stable at long-term follow up

AHI, apnea–hypopnea index; DBP, diastolic blood pressure; ESS, Epworth Sleepiness Scale; FOSQ, Functional Outcomes of Sleep Questionnaire; FU, follow-up; NR, not reported; SBP, systolic blood pressure; SpO₂, peripheral oxygen saturation.

In a pooled analysis of 518 patients from 45 studies, Zaghi et al. (2016) reported a mean AHI reduction of 80% following MMA (57.2–9.5) (14). A 2018 systematic review of 20 studies (*n* = 462) found a mean AHI decrease from ∼54–9.8 events/h (mean drop ∼44.8 events/h, ∼83%), with all studies achieving the surgical success threshold of >50% AHI reduction and postoperative AHI <20 (15).

### Summary of outcome metrics

#### Apnea–hypopnea index (AHI)

Meta-analyses consistently demonstrate ∼80% reduction in AHI (from ∼50–60 to <10 events/h) following MMA (Table 2) (14, 15).

#### Oxygen saturation

MMA significantly improves nocturnal oxygenation. In a systematic review of 518 patients, nadir SpO₂ rose from ∼70% to 87% (*p* < 0.001) (14). Likewise, Lye et al. found nadir SpO₂ increase from 76.5% to 85.0% after MMA (*P* = .0001) (22).

#### Daytime sleepiness

Patients report dramatic relief of sleepiness. In a meta-analysis summarizing data from 45 studies and 518 unique patients, Epworth Sleepiness Scale (ESS) scores fell from ∼13.5 to ∼3.2 (14). In another retrospective single center study analyzing outcomes in 265 patients who underwent MMA surgery, mean ESS scores improved from 12.9 to 5 (23).

#### Quality of life

Multiple studies show large gains in functional status. For example, in a prospective multicenter cohort study (*n* = 30, followed for a mean of 6.6 months after MMA), FOSQ (Functional Outcomes of Sleep Questionnaire, designed to capture the difficulty with daily activities such as concentration, watching television related to fatigue with higher scores indicating no difficulties) scores rose from 14.1 to 18.3 (*P* < .001) after MMA, with an average decrease of the AHI from 39.6 to 7.9 (24). Lye et al. found 93% of MMA patients achieved a clinically successful FOSQ score ≥18, with significant improvements in all domains (productivity, social function, vigilance, etc.) (22).

#### Effects on cardiovascular and metabolic markers

MMA not only improves OSA-specific outcomes but also translates into broader health benefits. Several studies have shown meaningful improvements in cardiovascular and metabolic parameters following surgery. These cardiometabolic improvements are particularly relevant within the context of healthy aging, where blood pressure control and reduced systemic inflammation are recognized determinants of longevity (25, 26).

#### Blood pressure

Islam et al. reported short-term data (*n* = 45) demonstrating a reduction in systolic blood pressure (SBP) from 131 to 127 mmHg (*p* < 0.001), with even greater improvements among hypertensive patients (SBP ↓6 mmHg, DBP ↓10 mmHg) (27). Boyd et al. confirmed these findings in a long-term cohort (*n* = 30, mean follow-up 6.6 years), showing sustained OSA control (AHI 49→10.9) and a persistent diastolic BP reduction from 83.7 to 79.0 mmHg (*p* < 0.05) (24, 28). By comparison, CPAP therapy typically lowers BP by only 2–3 mmHg, suggesting MMA may provide more robust cardiovascular benefits (29).

#### Body mass Index (BMI) and metabolism

A 2025 meta-analysis of 31 studies (*n* = 1,597) found a modest but significant reduction in BMI after MMA, with a mean decrease of 0.74 kg/m^2^ (from 29.15 to 28.25; 95% CI, −1.35 to −0.12; *p* = 0.02) (30). While data on glucose and lipid profiles remain limited, preliminary evidence indicates a trend toward improved systemic inflammation. For example, mean high-sensitivity C-reactive protein (hsCRP) declined from 2.6 to 2.3 mg/L, though this did not reach statistical significance (24).

#### Complications and risks

MMA was shown to have a favorable safety profile considering the physiologic effect size and ability to achieve successful AHI reduction and abovementioned benefits. Several studies have documented the safety and low morbidity of the procedure. In one of the largest systematic reviews, Holty et al. (627 patients) found that pooled major and minor complication rates were 1% and 3%, respectively, without perioperative deaths (14). A more recent systematic review performed by Walker et al. (31 studies, *n* = 1,597) confirmed the safety profile, and identified transient CN5 dysfunction (related to inferior alveolar nerve manipulation during BSSO), as the most common self-limiting sequela which typically resolves 6–12 months postoperatively (30). Other less common complications may include infection, malocclusion, worsening of facial appearance, hardware loosening, TMJ dysfunction and rare events of skeletal nonunion (13, 30).

#### Alternative therapies

CPAP remains the first-line therapy for patients with OSA, and it can be highly effective. However, real world adherence can be limited. More than 50% of patients are intolerant to CPAP and non-adherent patients have been shown to carry a 10% absolute increase in 5-year mortality compared with adherent patients, and nonadherence to CPAP was shown to be associated with an increase in 30-day hospital readmission rates (due to cardiovascular/pulmonary decline) (19, 31). Mandibular advancement devices (MADs) are able to reduce AHI and improve symptoms of sleepiness in patients who suffer from mild-to moderate OSA and are typically well tolerated (32). However, the ability to improve AHI is lower when compared to CPAP and MADs are generally inadequate for severe OSA. Furthermore, the devices are custom made to fit someone's dentition and thus require healthy teeth. These devices can lead to TMJ discomfort, dry mouth, gum irritation and can thereby limit patient tolerance (32).

Surgical alternatives include soft tissue procedures such as uvulopalatopharyngoplasty with or without tonsillectomy (in case of tonsillar hypertrophy) which have lower success rates (reported as low as 30% with average success rates at 60%–65%), high perioperative discomfort and possible morbidity and less long-term durability (12, 32). Other options target potential sites of obstruction at the level of the nose (turbinate reduction, septoplasty), Tongue (genioglossus advancements, tongue base reduction, upper airway stimulation with implantation of a hypoglossal nerve stimulator) (12, 32). However, compared to MMA (which allows correction of skeletal reasons for airway narrowing), reported success rates for alternatives are generally lower than the reported 80%–90% of MMA. Phase 1 surgery success is between 42% and 75% (12).

#### Aesthetic outcomes and considerations

For the plastic surgeon, the aesthetic consequences of MMA are inseparable from its functional indication, and special attention must be placed on maintaining or optimizing a balanced face. MMA reshapes the central facial complex, including nose, lips, chin, and their interrelationships, and the plastic surgeon's training uniquely positions them to plan these changes holistically. A meta-analysis by Jamal et al. found patient satisfaction with facial appearance of 94.2% following MMA, indicating that airway reconstruction and aesthetic optimization can be achieved concurrently when planning is deliberate (33).

One central consideration is the angle of the occlusal plane relative to the Frankfort horizontal, which strongly influences the lateral facial profile (34, 35). During MMA for OSA, the maxillomandibular complex is typically rotated counterclockwise (CCW), and this rotation is itself an aesthetic tool. The principle was first articulated by Rosen in 1993 for patients with severe mandibular micrognathia, in whom deficient vertical ramus height, a steep occlusal plane, and Class II convex profiles reflect posterior maxillary deficiency with anterior vertical excess (35).

CCW rotation normalizes this morphology by advancing the mandible forward and upward, producing large sagittal gains at pogonion: in Rosen's series, mean B-point advancement was 17 mm against only 10 mm at the first molar, demonstrating that rotation amplifies chin and lower-face projection well beyond what pure translation can achieve. This distinction becomes particularly consequential in OSA, where mandibular advancements exceeding 10 mm are typically required for meaningful retrolingual airway gain. Achieving equivalent movement by symmetric horizontal translation would necessitate proportionate maxillary advancement, with the well-described aesthetic drawbacks: paranasal fullness, thinning of the upper lip, excessive maxillary incisor show, nasal tip over-rotation, alar base widening, and an unnatural midface-forward appearance (36).

CCW rotation circumvents this problem by concentrating soft-tissue gain where it is aesthetically desirable at pogonion, labiomental fold, submental contour, while the posterior maxilla and molars move comparatively little. he same airway gain is therefore achieved with a smaller absolute translation vector, and the lower facial third is shortened rather than lengthened, preserving or improving facial proportions; the rotation can additionally correct excessive maxillary incisor show and lip incompetence. The steeper mandibular advancement vector toward the hyoid and tongue base also makes biomechanical sense for airway expansion and produces favorable jawline definition and cervicomental angle improvements. Finally, by flattening the occlusal plane, CCW permits greater effective mandibular advancement even when preoperative overjet is minimal, since the chin translates forward considerably more than the molars and occlusal contacts are preserved, allowing the surgeon to deliver the magnitude of advancement OSA correction demands without compromising occlusion or facial harmony (35, 36).

#### Basic science: senescence and cellular dysfunction in OSA

Recent basic-science work delineates how intermittent hypoxia (IH) drives cardiometabolic and neurocognitive morbidity (9). IH creates a HIF-α isoform imbalance (↑HIF-1*α*, ↓HIF-2*α*) that increases ROS production via HIF-1–dependent NOX2 induction while weakening HIF-2–mediated antioxidant programs (e.g., SOD2); the resulting increase in oxidative stress activates chemoreflexes, blunts baroreflexes, elevates sympathetic tone and blood pressure, and contributes to insulin resistance/type 2 diabetes and hippocampal NMDA-receptor dysfunction with cognitive decline (9). Multiple additional pathways of cell injury have been proposed and may involve increased cellular stress response, increased inflammation on cellular level, oxidative stress and cellular senescence (9). Some studies in particular suggest that OSA leads to premature cellular aging and cellular senescence – a consequence of chronic endogenous/exogenous stress exposure leading to dysfunctional cell populations that have a tendency to promote inflammatory/fibrotic and apoptotic processes. For example, preadipocytes exposed to intermittent hypoxia (IH) exhibited elevated mitochondrial reactive oxygen species (mtROS) and a higher fraction of cells with nuclear *γ*H2AX and p16^INK4a^—canonical markers of DNA damage and cellular senescence (9, 37). In cardiac tissues, it was shown that chronic IH can lead to cardiovascular inflammation as shown by increased NF-kB activation which was shown to be reversible by CPAP therapy (38). In several additional studies, it was shown that improvement or normalization of nocturnal oxygenation (e.g., via CPAP) is able to reverse or minimize some of the hypoxia/ROS riven cellular stress pathways and thus provide a mechanistic rationale for therapies (including MMA) that can improve oxygenation and downstream tissue injury (39, 40).

#### Long-term health and longevity markers

Long-term studies confirm that the benefits of MMA are durable. In the 6.6-year follow-up cohort by Boyd et al., mean AHI decreased from 49 preoperatively to 10.9 postoperatively, with more than 80% of patients maintaining an AHI ≤15 (24). Improvements in subjective sleepiness (ESS) and quality of life (FOSQ) observed shortly after surgery remained significant years later. Importantly, the early reductions in blood pressure also persisted, with diastolic BP remaining 4–5 mmHg lower compared to baseline (24).

Although no large trials have directly measured mortality after MMA, surgical series suggest excellent safety with extremely low all-cause mortality. In one comparative study of OSA patients and orthognathic surgery controls, no deaths were reported in either group (28 OSA patients included)^5^ For context, untreated sleep-disordered breathing, including severe OSA, is associated with a 2–4-fold increase in mortality compared with the general population (10). Thus, by eliminating nocturnal hypoxemia, reducing blood pressure, and restoring daytime alertness, MMA is expected to carry meaningful survival benefits, even though this has not yet been directly studied.

On a cellular level, enhanced oxygenation has downstream cellular effects, including improved mitochondrial function, reduced oxidative stress, and modulation of inflammatory pathways (9). Together, these mechanisms may help slow age-related decline, reinforcing the role of MMA as not only a corrective procedure for OSA but also as a potential contributor to extending healthspan and longevity (9, 37).

#### Long-Term stability and late relapse

The durability of MMA has been examined in several intermediate- and long-term cohorts. Boyd and colleagues followed 30 patients prospectively for a mean of 6.6 years and reported sustained reductions in AHI (49 → 10.9 events/h), with 83.4% of patients maintaining an AHI ≤15 and 46.7% an AHI <5 (see Table 2) (24). Furthermore, Camacho et al. showed in a meta-analysis that stable reductions are achieved both in the intermediate term (1–4 years, mean AHI 48.3–8.4) and long term (4—8 years, 65.8–7.7) with partial loss of effects >8 years (mean AHI rebound to approximately 23 events/h) at very long term follow up (41).

Skeletal relapse and soft-tissue remodeling likely contribute to this late decline. Navasumrit and colleagues evaluated skeletal and airway stability following MMA for OSA and demonstrated measurable relapse at two-year follow-up, with posterior movement of the maxilla, soft palate, and tongue and inferior displacement of the hyoid, resulting in an objective reduction in oropharyngeal airway volume (13). Importantly, however, polysomnographic improvements often remain stable despite these anatomic changes, suggesting that the clinical benefit of MMA is not solely dependent on absolute airway caliber.

#### Cost-Effectiveness

The cost-effectiveness data on MMA is less well documented than the clinical benefit, however available data supports its value in appropriately selected patients. In a lifetime semi-Markov model, Tan and colleagues compared no treatment, CPAP only, and CPAP followed by surgery for CPAP-intolerant patients, and found that the surgical strategy was highly cost-effective (42). Surgical strategies added 0.265 quality-adjusted life-years over CPAP alone at an incremental cost-effectiveness ratio of $10,421/QALY, well below the commonly references U.S. willingness-to-pay threshold of $50,000–100,000/QALY (43). Much of the gain was driven by avoidance of cardiovascular events and motor vehicle collisions associated with untreated OSA. An important consideration is the fact that more than half of CPAP using patients are non-adherent and non-adherence comes at health cost, increased mortality and it is likely that MMAs higher upfront cost is offset by sustained and efficient reductions in AHI and cardiometabolic benefits as well as the elimination of CPAP-related expenses (43, 44). Direct economic comparisons between MMA and alternatives such as hypoglossal nerve stimulation are lacking and represent an important gap in the literature.

## Research gaps, future directions and conclusion

Despite robust evidence supporting MMA as the most effective non-tracheostomy surgical option for OSA, several controversies and unresolved questions remain. The optimal sequencing of surgery continues to be debated by some surgeons. The historical Stanford protocol positioned MMA as a phase II procedure reserved for patients failing soft-tissue surgery (12). Nowadays a ’surgery/MMA first' approach or DISE-guided approaches propose to directly proceed to MMA in selected patients with skeletal factors or complete lateral pharyngeal wall collapse.

Furthermore, the relationship between magnitude of advancement, counterclockwise rotation, gain in airway (naso-,oro-,hypopharynx) volume and AHI reduction remains incompletely defined and a topic of ongoing research (20, 45). Recent volumetric analyses suggest this correlation is nonlinear, indicating that soft-tissue tensioning and three-dimensional vector pull may drive changes and a clearer dose-response assessment is needed (20, 45). As mentioned earlier in this manuscript, some of the older literature used 2D cephalograms to estimate degree of advancement and airway change (PAS). The movements are done in 3D space and a simple 2D measurement may not account accurately reflect other 3D changes such as the degree of CCW, change at the genial tubercle, change in airway. Further studies are needed to improve our understanding of what truly shapes and expands the posterior airway (degree of CCW combined with changes at A/B point correlated with volumetric assessment of specific sub-airway regions.

Beyond these clinical questions, the evolving landscape of surgical techniques itself warrants consideration as an area of ongoing development. Advances in virtual surgical planning (VSP), intraoperative navigation, and patient-specific three-dimensional printing have fundamentally transformed the precision with which osteotomies can be designed and executed in MMA. These technologies enable simulation-based preoperative optimization of skeletal vectors, counterclockwise rotation angles, and soft-tissue tension profiles prior to any incision being made. Importantly, they have also facilitated the emergence and refinement of modified and combined osteotomy concepts, such as segmental Le Fort I variants, interpositional grafting strategies, or hybrid MMA-distraction protocols — that extend the surgical envelope beyond conventional bimaxillary advancement. In carefully selected patients, these modifications may allow individualized airway augmentation across specific pharyngeal compartments while simultaneously accounting for aesthetic and occlusal constraints. The integration of VSP with cone-beam CT-based airway volumetry and finite-element modeling of soft-tissue displacement represents a particularly promising methodological frontier, one that may ultimately allow a more mechanistically grounded, patient-tailored approach to skeletal advancement planning. As this technological infrastructure continues to mature, it is likely to generate both novel osteotomy paradigms and more granular outcome data, thereby directly addressing several of the evidence gaps outlined above.

Third, very-long term data is lacking. One of the leading studies was performed by Camacho et al. in 2019 and showed partial relapse >8 years after MMA. Longer follow up data and larger cohort studies are needed to assess late skeletal relapse, soft-tissue remodeling (and specifically the anatomic compartments), durability of AHI control and the long-term impact of MMA on facial aesthetics and airway reconstruction (46).

Finally, comparative cost-effectiveness and clinical effectiveness studies between MMA and other modalities (e.g., hypoglossal nerve stimulation) are lacking and would help guide clinical decision making in selected patients. Addressing the gaps through prospective studies, long-term registries and direct comparative trials will be essential to refine the role of MMA in the broader landscape of procedures available to OSA management.
